# Elevated plasma levels of NT-proBNP in ambulatory patients with peripheral arterial disease

**DOI:** 10.1371/journal.pone.0253792

**Published:** 2021-07-21

**Authors:** Bader Alsuwailem, Abdelrahman Zamzam, Muzammil H. Syed, Elisa Greco, Mark Wheatcroft, Charles de Mestral, Mohammed Al-Omran, John Harlock, John Eikelboom, Krishna K. Singh, Rawand Abdin, Mohammad Qadura

**Affiliations:** 1 Department of Surgery, University of Toronto, Toronto, ON, Canada; 2 Division of Vascular Surgery, St. Michael’s Hospital, Toronto, ON, Canada; 3 Department of Surgery, Hamilton General Hospital, Hamilton, Canada; 4 Population Health Research Institute, McMaster University, Hamilton, Ontario, Canada; 5 Department of Medicine, McMaster University, Hamilton, ON, Canada; 6 Department of Medical Biophysics, Schulich School of Medicine and Dentistry, University of Western Ontario, London, ON, Canada; 7 Keenan Research Centre for Biomedical Science, Li Ka Shing Knowledge Institute of St. Michael’s Hospital, Toronto, ON, Canada; Scuola Superiore Sant’Anna, ITALY

## Abstract

N-terminal pro B-type natriuretic peptide (NT-proBNP), a cardiac disease biomarker, has been demonstrated to be a strong independent predictor of cardiovascular events in patients without heart failure. Patients with peripheral arterial disease (PAD) are at high risk of cardiovascular events and death. In this study, we investigated levels of NT-proBNP in patients with PAD compared to non-PAD controls. A total of 355 patients were recruited from outpatient clinics at a tertiary care hospital network. Plasma NT-proBNP levels were quantified using protein multiplex. There were 279 patients with both clinical and diagnostic features of PAD and 76 control patients without PAD (non-PAD cohort). Compared with non-PAD patients, median (IQR) NT-proBNP levels in PAD patients were significantly higher (225 ng/L (120–363) *vs* 285 ng/L (188–425), p- value = 0.001, respectively). Regression analysis demonstrated that NT-proBNP remained significantly higher in patients with PAD relative to non-PAD despite adjusting for age, sex, hypercholesterolemia, smoking and hypertension [odds ratio = 1.28 (1.07–1.54), p-value <0.05]. Subgroup analysis showed elevated NT-proBNP levels in patients with PAD regardless of prior history of CHF, CAD, diabetes and hypercholesteremia (p-value <0.05). Finally, spearmen’s correlation analysis demonstrated a negative correlation between NT-proBNP and ABI (ρ = -0.242; p-value < 0.001). In conclusion, our data shows that patients with PAD in an ambulatory care setting have elevated levels of NT-proBNP compared to non-PAD patients in the absence of cardiac symptoms.

## 1. Introduction

Peripheral artery disease (PAD) is a chronic cardiovascular condition that is more prevalent with aging. The prevalence of PAD is increasing, reflecting growing prevalence of cardiovascular risk factors and increasing survival of patients with PAD [1–3]. Patients with PAD usually have multiple vascular beds affected with atherosclerosis, such as the coronary and carotid arteries [4]. As a result, patients with PAD suffer from a high risk of cardiovascular events and mortality [3,5].

The association between PAD and coronary artery disease is well established [6]. Progressive PAD has been shown to be an independent predictor of coronary and cerebrovascular morbidity and mortality [7]. Similarly, an abnormal ankle-brachial index (ABI) confers a higher risk of cardiovascular mortality [8–10]. Despite the strong association between PAD and cardiovascular morbidity, cardiovascular risk factors in patients with PAD are less intensively managed than in patients with coronary artery disease [11]. Unsurprisingly, this inadequacy of medical management within patients with PAD is associated with increased risk of major adverse cardiovascular events.

Plasma B-type natriuretic peptide (BNP) is an extensively studied cardiac marker [12,13]. It is synthesized by ventricular myocytes and released into circulation in response to myocardial stretch [14]. N-terminal pro-BNP (NT-proBNP) is a 76 amino-acids long protein that is a biologically inert by-product of BNP [15,16]. NT-proBNP can be used to diagnose patients with left ventricular dysfunction and congestive heart failure and is a valuable tool for determining prognosis and evaluating response to therapy [17–19]. In particular, several studies have established 125ng/L as a reference limit for the upper normal levels of NT-proBNP in non-acute settings to help improve the specificity of diagnosing patients with heart failure [20,21]. Furthermore, NT-proBNP predicts cardiovascular events such as myocardial infarction, stroke and all-cause mortality in patients without heart failure [22,23]. Since NT-proBNP is also elevated in patients with stable cardiac disease [24], this study aims to assess the status of NT-proBNP in ambulatory patients with PAD in the absence of acute coronary artery disease and acute congestive heart failure.

## 2. Materials and methods

### Ethics approval

This study was approved by the Unity Health Toronto Research Ethics Board at St. Michael’s Hospital—University of Toronto, Ontario, Canada (#16–375, 8 February 2017). Written and verbal informed consent was obtained from all participants. Furthermore, all methods were carried out in accordance with the relevant guidelines and regulations.

### Patient selection

Patients presenting consecutively to vascular surgery ambulatory clinics at St. Michael’s Hospital between February 2019 and February 2020 were recruited for this study. Patients with PAD were defined based on an ABI < 0.9 and abnormal distal pulses examination with or without claudication. A control group of patients without PAD were also recruited. This control group of patients were defined as patients with ABI ≥ 0.9, palpable distal pulses, and no clinical history of claudication with at least one cardiovascular risk factor (smoking, hypertension, hyperlipidemia, diabetes, smoking or prior history of coronary arterial disease. The TBI (toe brachial index) measurements were performed when the ABI values could not be calculated due to non-compressible tibial vessel. Patients with TBI < 0.67 were characterized as having PAD, whereas controls had a TBI of ≥ 0.67. Patients that met any of the following criteria were excluded from this study: 1) patients with renal disease (chronic kidney disease stages 3, 4 and 5), 2) patients with acute limb ischemia, 3) patients with 12-month history of acute coronary syndrome (ACS), established diagnosis of congestive heart failure (CHF) stages I-IV or uncontrolled arrhythmia, and 4) patients with elevated troponin levels.

### Baseline measurements

Baseline variables were defined as previously described [25]. Patients with a glycosylated hemoglobin A1c ≥6.5% or using anti-diabetic medication were classified as having type 2 diabetes mellitus (DM). Patients on anti-hyperlipidemic medication or having a total cholesterol >5.2 mmol/L or triglyceride >1.7 mmol/L were classified as individuals with hypercholesterolemia. Patients using antihypertensive medication or having a systolic blood pressure ≥130mmHg or a diastolic pressure ≥80 mm Hg were classified as individuals with hypertension. Renal disease was defined as estimated glomerular filtration rate less than 60 mL/min/1.73 m2. In addition to the baseline measurements mentioned above, previous history of stroke, transient ischemic attack (TIA), and smoking status was also recorded for each patient.

### Sample processing and NT-proBNP multiplex assay

Patient blood samples were drawn into EDTA-containing vacutainer tubes, with plasma being extracted *via* centrifugation at 3000 rpm for 10 min 4°C. These plasma samples were then aliquotted and stored at −80°C.

To determine the concentrations of NT-proBNP levels, plasma samples were analyzed in duplicate using MILLIPLEX MAP Human Cardiovascular Disease (CVD) Magnetic Bead Panel 1 (EMD-Millipore; Billerica, MA). Prior to any sample analysis, Fluidics Verification and Calibration bead kits (Luminex Corp) were used to calibrate the MagPix analyzer (Luminex Corp; Austin, Texas). 50 beads or more for NT-proBNP were acquired using Luminex xPonent software and analyzed using Milliplex Analyst software (v.5.1; EMD-Millipore). To prevent any inter-assay variability, all sample analyses were carried out on the same day. Sample intra-assay Coefficients of Variability (CV) was <10% while the inter-assay CV was 15%. The calculated limit of detection (LOD) for our assay was 59 ng/L, whereas the limit of quantification (LOQ) was 181ng/L.

To establish a normal reference range for NT-proBNP using MILLIPLEX MAP Human Cardiovascular Disease (CVD) kit in human samples, we recruited 20 young healthy individuals without a previous history of cardiovascular risk factors or use of medication (S1 Table). The normal range for NT-proBNP was established by calculating the 99th percentile of NT-proBNP levels within this healthy patient population, which amounted to 187 ng/L.

### Statistical analysis

Patients with PAD were compared to non-PAD controls. Data are presented as mean ±SD or as median and interquartile ranges (IQR). Assessment of normality was done using Shapiro-Wilk test. Since the continuous variables were not normality distributed, Mann-Whitney U test was used to evaluate the collected data, while Kruskal-Wallis test was used to determine any significant differences between two or more independent groups. Post-hoc test was used for pairwise comparisons to further analyze these differences. Fisher’s exact test or chi-square test was used for categorical variables analysis. Patients with PAD were stratified into subgroups based on their ABI as per the Society for Vascular Surgery’s wound, ischemia, and foot infection (WIfI) classification system [26]. Logistic regression was used to calculate the odds ratios (ORs) with 95% confidence intervals (95% CI) for the development of PAD given one standard deviation increase in NT-proBNP levels. Z-scores NT-proBNP values were used for ease of odds ratio interpretation. Multiple logistic regression analyses were used to examine the association of NT-proBNP and other parameters with the development of PAD. Correlations between PAD and NT-proBNP were analyzed using Spearman’s correlation. All study hypothesis testing was carried out at the 5% (2-sided) significance level. All data were collected using Microsoft Excel sheet, analysis was carried out using SPSS software version 23 (SPSS Inc., Chicago, Illinois, USA), whereas figures were constructed using GraphPad Prism version 8.0.0.

## 3. Results

### Cohort description

A total of 355 consecutive participants were enrolled in this study, of whom 279 (79%) were patients with PAD and 76 (21%) were non-PAD controls. To note due to non-compressible (NC) vessels, there were 24 patients with abnormal ABI values. In such cases, the TBI was used to classify patients as either PAD or non-PAD. All 24 patients with NC vessels had TBI values < 0.67, (TBI = 0.33 ±0.126). Mean age was 68 years, and 66% were males (Table 1). There were no significant baseline differences between the PAD and non-PAD patients apart from age and ABI. Table 1 presents the baseline characteristics of both patient groups.

**Table 1:** Patient demographics and clinical characteristics.

	Non-PAD (n = 76)	PAD (n = 279)	p-value
Age (Years)			
Mean (SD) †	61 (15)	70 (10)	0.004
ABI at collection			
Mean (SD) †	1.05 (0.09)	0.60 (0.21)	0.001
Frequency (%) ‡			
Sex (Male)	51 (67)	182 (66)	0.790
Hypertension	50 (66)	212 (77)	0.058
Hypercholesterolemia	49 (65)	208 (76)	0.052
Diabetes	36 (47)	140 (50)	0.664
Smoking	56 (74)	231 (83)	0.076
Stroke/transient ischemic attack	22 (29)	87 (31)	0.708
Chronic Congestive heart failure	0 (0)	13 (5)	0.055
Coronary artery disease	33 (43)	110 (39)	0.529

†differences between groups were compared using Mann-Whitney test.

‡ differences between groups were compared using chi-square test.

means and standard deviations were calculated for continuous variables.

frequencies and percentages were calculated for categorical variables.

### NT-proBNP levels in patients with PAD

**Fig 1:**
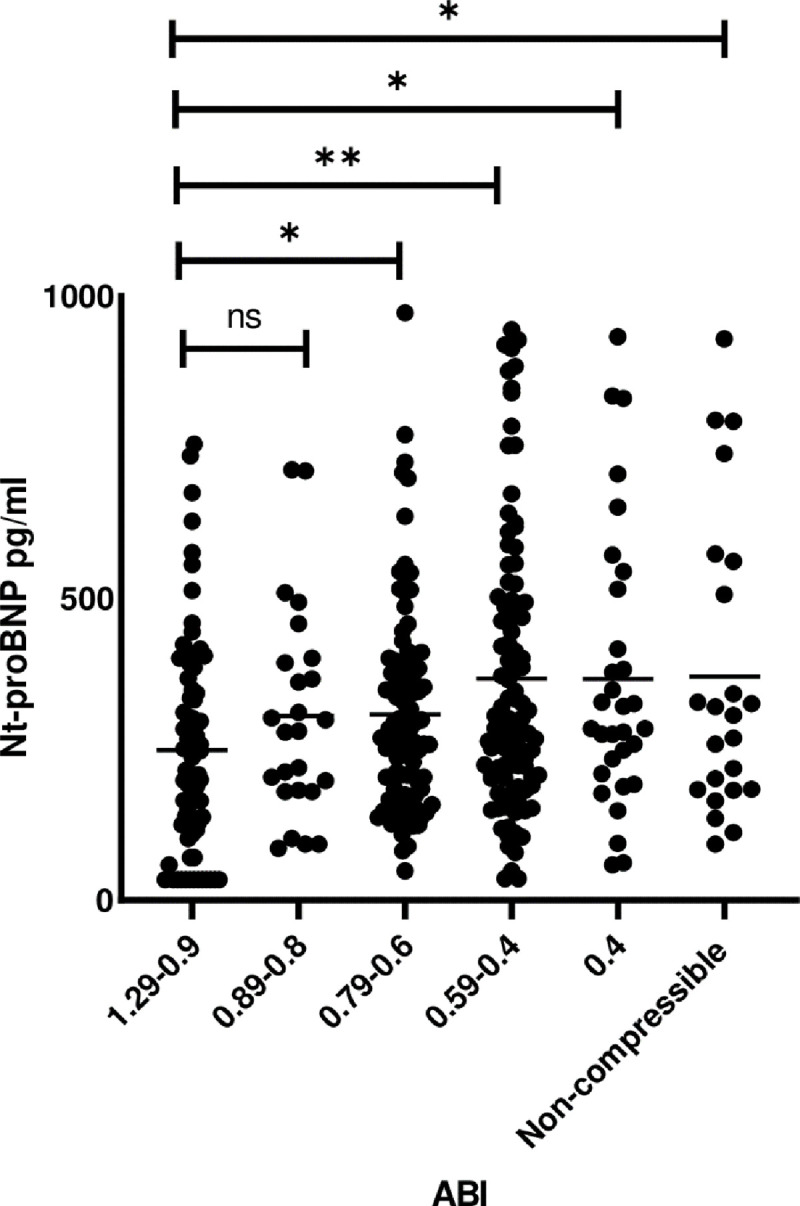
NT-proBNP levels in controls and PAD patients stratified based on their ABI as per the WIfI tissue ischemia classification system. Scattered plot representing median NT-proBNP (ng/L) plasma levels among controls and patient with PAD. PAD patients were stratified based on ABI as per WIfI classification system. * p-value < 0.05. ** p-value < 0.0001.

Circulating levels of NT-proBNP in the PAD group were significantly higher than in the control group (median = 285 ng/L; IQR 188–425 vs median = 225 ng/L; IQR 120–363, p- value = 0.001, respectively). Furthermore, to investigate the trend of NT-proBNP levels in PAD, patients were stratified based on their ABI as per the WIfI tissue ischemia classification system. Compared to non-PAD patients, significant elevation of NT-proBNP levels were observed mainly in patients with ABI ≤0.79- mild, moderate and severe ischemia (Fig 1). We also noted elevated NT-proBNP levels in PAD patients with NC vessels. However, no statistical difference in NT-proBNP levels were noted among PAD patients. Therefore, relative to controls, our data suggest that NT-proBNP levels are only statistically significant in PAD patients with worsening tissue ischemia (ABI ≤0.79). In further analysis, the association between NT-proBNP and ABI was investigated via a spearmen correlation test within our patient population. Our data demonstrates a significant negative correlation between NT-proBNP and ABI (ρ = -0.242; p-value < 0.001).

### Influence of confounding factors on NT-proBNP levels in patients with PAD

To adjust for potential NT-proBNP confounding factors in patients with PAD, multiple stepwise regression was conducted (**Table** 2). Overall, NT-proBNP was independently associated with PAD (unadjusted OR 1.35 (95% CI: 1.14–1.61)). Table 2 highlights additional models that demonstrate a significant association between NT-proBNP levels and patients with PAD after adjusting for additional cardiovascular factors.

**Table 2:** Influence of individual factors on the odds ratios for PAD per one standard deviation increase in plasma NT-proBNP levels.

Regression models	Odds ratio (95% CI) for PAD†
**Unadjusted**	1.35 (1.1–1.6) *
**Model 1 (**adjusted for age and sex)	1.30 (1.09–1.56) *
**Model 1 + Hypercholesteremia**	1.30 (1.09–1.56) *
**Model 1 + Hypercholesteremia + Smoking**	1.28 (1.07–1.54) *
**Model 1 + Hypercholesteremia + Smoking + Hypertension**	1.28 (1.07–1.54) *

† Binary logistic regression models for PAD per one standard deviation increase in NT-proBNP levels (ng/L).

* p-value < 0.05.

### Subgroup analysis

In order to better understand the relationship between the levels of NT-proBNP in patients with PAD, we conducted a subgroup analysis looking at potential cardiovascular confounding factors (prior history of CHF, prior history of CAD, diabetes and hypercholesteremia–all of which have previously been shown to be associated with NT-proBNP [22,27–32]). Here, levels of NT-proBNP were compared among PAD patients with/without confounding factor against non-PAD controls (Table 3). Our analysis shows that PAD patients have higher levels of NT-proBNP than non-PAD patients, irrespective of prior history of CHF or CAD, hypertension, diabetes and hypercholesteremia.

**Table 3:** Median and interquartile ranges for NT-proBNP levels among PAD patients with and without cardiovascular risk factors.

Condition	PAD *(condition absent)*	P value†	PAD *(condition present)*	P value†
**Hypercholesterolemia**	301 (204–401)	0.017	284 (184–454)	0.004
**Diabetes**	284 (201–425)	0.004	288 (176–438)	0.011
**Prior history of chronic congestive sports failure**	284 (188–418)	0.003	341 (201–668)	0.014
**Prior history of coronary artery disease**	288 (190–391)	0.009	279 (181–493)	0.004

† p-value compared to non-PAD controls.

## Discussion

We demonstrated that circulating levels of NT-proBNP are elevated in ambulatory outpatients with PAD compared with controls who do not have PAD. Relative to non-PAD, our data suggests that NT-proBNP levels remain to be elevated in patients with PAD despite adjusting for potential confounding factors. Subgroup analysis demonstrated elevated levels of NT-proBNP primarily in PAD patients with ABI ≤0.79. Lastly, our data show a positive association between PAD and NT-proBNP in spearmen correlation studies as well as subgroup analysis.

Patients with PAD usually have atherosclerosis in other vascular beds such as the coronary and carotid arteries [33]. It has been established that PAD is a strong prospective predictor of cardiac and cerebrovascular disease morbidity and mortality [34]. This puts PAD patients at a high risk of cardiovascular events and mortality. Unfortunately, several studies suggest that patients with PAD do not receive similar cardiovascular risk stratification as compared to patients with coronary artery disease [11]. This often delays the initiating of medical management and therefore, increases the risk of cardiovascular events in patients with PAD [35–37]. NT-proBNP has been shown to associated with cardiovascular events and all-cause mortality in an unselected, large population of elderly patients, in addition to patients with acute coronary syndrome or congestive heart failure [38]. In this study, we demonstrated that NT-proBNP is elevated in patients with PAD. This finding complements previously published literature on the strong association between PAD and increased cardiovascular events and mortality [39]. Interestingly, NT-proBNP remained to be elevated in patients with PAD despite adjusting for cardiovascular risk factors–akin to other published findings on patients with stable ischemic heart disease as well as patients with stable heart failure [24,40]. Therefore, our data presents an association between NT-proBNP and patients with PAD. Subgroup analysis demonstrated that patients with reduced ABI < 0.79 primarily have significantly elevated NT-proBNP levels–putting them higher risk of future cardiovascular events. Thus, this patient subgroup might benefit from more intensified cardiovascular risk stratification.

It is worth pointing out that our non-PAD control group had higher levels of NT-proBNP compared to healthy control patient populations from other studies [41]. Several reasons may explain this discrepancy of NT-proBNP levels. First, the detection method used in this study (Milliplex MAP) to measure NT-proBNP differs from the detection method (Roche ECLIA) used in clinical practice–resulting in different NT-proBNP values [42]. For instance, the normal reference range of NT-proBNP in healthy patients (no prior cardiovascular risk factors) using our detection method was calculated to be 187 ng/L. This reference value differs from the normal reference values used in clinical guidelines (NT-proBNP <125ng/L) [20]. Different detecting methodologies resulting in varying NT-proBNP levels among control groups has previously been suggested in the literature [27,43]. Second, our control group comprised of patients with at least one cardiovascular risk factor as a non-PAD control group, in order to account for possible confounding. However, other studies opted to utilize healthy patients who were free from cardiovascular diseases and have denied the use of medication as their control patients [41]. Thus, the difference in control patients may also partially explain the discrepancy between NT-proBNP levels observed in this study versus other studies [41]. With that being said, it is important to note that the purpose of this study was to demonstrate a correlation between levels of NT-proBNP and PAD disease status, which was adequately achieved within the current study design and methodology utilized.

The elevated NT-proBNP levels PAD patients might be explained by an underlying, undiagnosed cardiac disease (e.g. asymptomatic LV dysfunction, heart failure etc.) which has not been screened for, or yet to be established. Therefore, by selecting a subgroup of PAD patients (e.g. those with advanced PAD) physicians may potentially intervene by advanced cardiac screening, referral to cardiologists and further intensifying patients’ medical therapy. Discussing the clinical relevance of enchaining cardiovascular risk stratification in patients with PAD based on NT-proBNP levels is beyond the scope of this paper. However, recent evidence recommends screening of the general population at high risk of major adverse cardiovascular events using NT-proBNP [44,45]. Within the PAD population, patients with advanced PAD (i.e. “reduced ABI”) would appear to benefit from NT-proBNP screening the most in order to reduce their risk of cardiovascular events. However, larger studies are needed to validate the findings presented in this paper.

The interpretation of NT-proBNP levels in asymptomatic patients has been previously, validated, and cut off points have been established to help physicians stratify the cardiovascular risk in patients. The cut-off value for an abnormal outpatient testing for NT-proBNP is different than the threshold within acute clinical settings. For instance, an abnormal NT-proBNP in an ambulatory setting was reported to range from 100–150 ng/L in heart failure patients [46]. However, a meta-analysis conducted by Rodseth et al. showed that a preoperative NT-proBNP value of ≥ 300 ng/L was associated with an increased risk of the primary outcome (i.e., death or nonfatal myocardial infarction) at 30 days after noncardiac surgery [47]. In our study, we observed elevated levels of NT-proBNP with a median of 285 ng/L among patients with PAD. This means that almost 50% of the recruited PAD patients had NT-proBNP levels above the threshold of 300ng/L which is associated with an increased risk of the primary outcome in a preoperative setting for noncardiac surgery patients. Based on our obtained data, we would like to clarify that we do not recommend routine testing of NT-proBNP among all ambulatory patients with PAD; however, we wanted to demonstrate that the levels of NT-proBNP are significantly elevated in ambulatory patients with PAD (especially advanced PAD). Large clinical trials are needed to confirm these findings. Therefore, our data suggests that patients with PAD might have concomitant cardiovascular diseases such as LV dysfunction that has not been previously established. This paper also sheds more light on the importance of aggressive medical therapy in PAD patients. As previously shown by McDermott et al, patients with PAD are usually less medically optimized as their CAD counterparts, despite patients with PAD potentially having a concomitant undiagnosed cardiac disease such as LV dysfunction [11].

This study has several potential limitations. Due to this being a cross-sectional study, there might be some inherent selection and information bias. Another limitation is that ABI was calculated utilizing the highest ankle pressure at rest which may underestimate the atherosclerotic burden. However, significant pressure differences at rest are more pronounced in multilevel peripheral vascular disease, reflecting a higher burden of atherosclerosis. Furthermore, the levels of NT-proBNP observed in this study may be influenced by sex and age, as suggested by the literature [48–50]. Since the incidence of PAD rises significantly with increased age, it is not surprising that we observed higher values for older patients with PAD relative to patients without PAD. This could act as a potential confounding factor for the observed NT-proBNP levels in patients with PAD. Although our analysis controlled for confounding, the potential influence of age on NT-proBNP should be considered when interpreting the study findings. Lastly, there was no follow-up on the patients enrolled in this study. Perhaps following up on PAD patients over time and measuring their mortality and cardiovascular event rates outcomes would have informative.

## 5. Conclusions

In summary, our data demonstrates a significant association between patients with PAD and elevated levels of NT-proBNP, with this finding most pronounced in advanced PAD patients. Due to the strong association between high NT-proBNP and cardiovascular events, we believe that this subgroup of patients could potentially benefit from further intense cardiovascular risk factor modification and follow-up.

## Supporting information

S1 Table Normal reference range patient group demographics and clinical characteristics. (DOCX)
