# Supplementary material for: Elevated plasma levels of NT-proBNP in ambulatory patients with peripheral arterial disease
Source: PLoS One. 2021 Jul 21;16(7):e0253792. doi: 10.1371/journal.pone.0253792 (PMC8294530; doi:10.1371/journal.pone.0253792)
Supplement: S1 Table — (DOCX) [file pone.0253792.s001.docx]

Supplementary table 1: Normal Reference Range Patient Group Demographics and clinical characteristics.

| Demographics and Clinical Characteristics | Non-PAD (n= 20) |
| --- | --- |
| Age (Years)  Mean (SD) † | 31 (5) |
| ABI at collection  Mean (SD) † | 1.1 (0.06) |
| Sex (Male) | 55 % (11 ) |
| Frequency (%) | |
| Hypertension | 0 |
| Hypercholesterolemia | 0 |
| Diabetes | 0 |
| Smoking | 0 |
| Stroke/ transient ischemic attack | 0 |
| Chronic Congestive heart failure | 0 |
| Coronary artery disease | 0 |
